# A case of cough syncope diagnosed by the Valsalva maneuver and cough induction test under invasive arterial pressure measurement

**DOI:** 10.1002/ccr3.8798

**Published:** 2024-04-23

**Authors:** Yoshiro Kubota, Katsunori Okajima, Yuichi Nagamatsu, Tomoyuki Nakanishi, Takeaki Shirai, Makoto Kadotani, Yoshio Ohnishi

**Affiliations:** ^1^ Division of Cardiology Kakogawa Central City Hospital Kakogawa Hyogo Japan

**Keywords:** cough induction test, cough syncope, invasive arterial pressure measurement, Valsalva maneuver

## Abstract

Following the loss of consciousness during the Valsalva maneuver and cough induction test, real‐time arterial pressure measurement could clarify the significant blood pressure decrease in a patient with cough syncope.

## 1 INTRODUCTION

Reflex syncope is the most typical type of syncope. It includes vasovagal, carotid sinus, and situational syncope. Situational syncope is triggered by coughing, swallowing, defecation, and urination.^1^ The approach to diagnosing reflex syncope is to obtain a detailed medical history, exclude syncope due to other causes, such as cardiogenic syncope, and perform aggressive diagnostic procedures, such as the head‐up tilt test. Here, we report the case of a patient diagnosed with cough syncope.

## 2 CASE HISTORY/EXAMINATION

This case of cough syncope involved a 73‐year‐old man with obesity (body mass index [BMI]: 29.6 kg/m^2^) and a history of cough asthma. He has been prone to losing consciousness several times whenever he had a coughing fit, including twice in traffic accidents that occurred while driving in the last 4 years. An electroencephalogram was obtained, but the cause could not be identified. The patient was subsequently admitted to the hospital.

The patient was a past smoker and occasionally consumed alcohol. On cardiac auscultation, the heart sounds were clear and the rhythm was regular. The lungs were clear upon bilateral auscultation. Physical examination failed to reveal obvious jugular venous pressure elevation or lower extremity edema. There were no obvious problems with blood count or coagulation. Biochemistry showed mild renal impairment (creatinine, 1.19 mg/dL (normal 0.65–1.07 mg/dL); glycated hemoglobin, 7.0%). Electrocardiography revealed a sinus rhythm and premature atrial contractions, whereas chest radiography and computed tomography showed no abnormal findings. No arrhythmia was detected in the monitor electrocardiogram during hospitalization. Cardiac echocardiography did not reveal aortic stenosis or structural heart disease. The Schellong test for orthostatic hypotension was negative. Carotid echocardiography showed no plaque or stenosis, and blood pressure pulse wave analysis showed no significant blood pressure difference between the right and left arms that could suggest subclavian steal syndrome.

## 3 METHODS

In this unique case, cough‐related syncope was suspected based on the patient's medical history. When the Valsalva maneuver and cough induction test under electrocardiographic monitoring was performed in the sitting position at the bedside, the patient temporarily lost consciousness, and his radial artery pulse became unpalpable. No bradyarrhythmia was observed. He did not always lose consciousness when he had mild coughs. In addition, he had deep inspiration before coughing when he had syncope. Therefore, it was difficult to measure his blood pressure in real‐time, but the patient's syncope was affecting his activities of daily living, including causing car accidents. Hence, a definitive diagnosis was necessary, even if invasive tests were necessitated. We conducted a cough induction test under invasive arterial pressure measurement using a radial artery line and electrocardiography monitoring to monitor blood pressure later. We explained the complications associated with the procedure, such as puncture site bleeding, hematoma, nerve damage, and arteriovenous fistula, and obtained informed consent. The test was initiated by positioning the patient in a sitting position on the bed and placing a 20G intravenous catheter in his left radial artery.

## 4 CONCLUSION AND RESULTS

**FIGURE 1:**
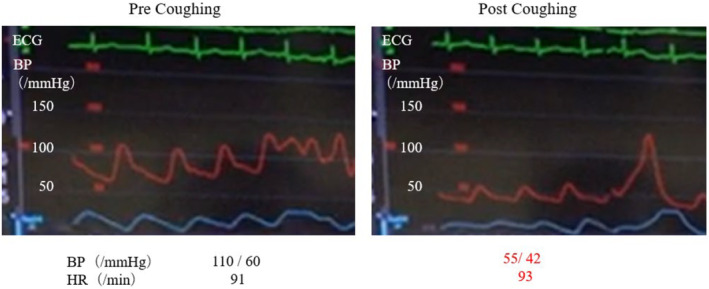
A cough induction test using a radial artery line to monitor blood pressure under invasive arterial pressure measurement. After instructing the patient to cough, the systolic blood pressure drastically decreased from 110 mmHg to 50 mmHg with loss of consciousness.

After the patient was instructed to cough, the systolic blood pressure drastically decreased from 110 mmHg to 50 mmHg with loss of consciousness. Therefore, hypotension due to coughing was diagnosed as the cause of syncope. No reflex bradyarrhythmia was observed, even when blood pressure decreased (Figure 1, Video S1).

For treatment, we provided lifestyle guidance that involved managing obesity with weight loss and cleaning the house to avoid allergenic substances. Moreover, the patient was prescribed a fluticasone/formoterol inhaler for cough asthma and vonoprazan for gastroesophageal reflux disease. Notably, the patient was also instructed to refrain from driving.

The patient's BMI remained unchanged during the 3‐month follow‐up at the outpatient clinic after discharge. However, no further fainting events occurred. We concluded that cough syncope could be clinically prevented by controlling the cough. However, when the Valsalva maneuver was performed in the consultation room, the radial arterial pulse became unpalpable, and the patient's eyes rolled into his head. Therefore, we continued to provide weight loss guidance for obesity management.

## 5 DISCUSSION

Men in their 30s and 50s who are obese or heavy smokers are susceptible to cough syncope.^2^ The condition is caused by increased intrathoracic pressure, which reduces venous return and cardiac output and increases intrathoracic pressure, cerebrospinal cord pressure, and cerebral blood volume.^3^ It is well established that the vagal reflex is triggered by baroreceptor and carotid artery hypersensitivity.^4^ In this case, blood pressure dramatically decreased during the Valsalva maneuver and cough induction test; however, bradycardia was not observed. Decreased venous return and increased cerebrospinal pressure after an elevated intrathoracic pressure induced by coughing associated with cough asthma are thought to contribute to syncope. In addition, the heart rate should rise reflexively; however, it is thought that the vagal reflex caused by coughing suppressed the increase in heart rate, resulting in only a slight increase. Therefore, treating underlying disorders and lifestyle guidance, such as weight loss for obesity, are the basic approaches to resolving the condition.^5^ There have been reports of pacemaker implantation in patients with refractory syncope and abnormal electrocardiograms; the pacemaker was implanted for carotid sinus hypersensitivity with mixed cardioinhibitory and vasodepressor responses.^6^ These cases had atrioventricular nodal abnormalities that caused the syncope, which was not observed in the present case. In a previous study, an antitussive drug was administered to control cough, and lansoprazole was administered to patients with gastroesophageal reflux disease to improve their condition.^7^ Vonoprazan used in this case is a potassium‐competitive acid inhibitor that inhibits H^+^‐K^+^ ATPase without depending on intragastric pH and is expected to suppress gastric acid secretion more rapidly and sustainably than the conventional proton pump inhibitor.^8^

The Valsalva maneuver can decrease blood pressure during cough syncope. Among the four‐phase reactions during which blood pressure fluctuates, a decrease is observed in phases II and III.^9^, ^10^ Cardiac output and blood pressure can be lowered after a decrease in venous return because of continued breathing during phase II. In phase III, the blood pressure is lowered owing to the decrease in intrathoracic pressure immediately after the discontinuation of swelling and blood inflow into the pulmonary vascular bed.

At the evaluation after discharge from the hospital, the Valsalva procedure in the consultation room resulted in a loss of palpability of the radial artery pulse and sursumvergence. This observation implies that the syncope could occur without coughing. It might have been better to perform a head‐up tilt test to evaluate neurally mediated syncope. However, it was not strongly suspected based on the patient's medical history. Allowing the patient to resume driving can cause difficult problems. Therefore, we showed him the result of drastic pressure decrease just after coughing following his loss of consciousness, and he was convinced to accept our advice to stop driving.

To our knowledge, this is the first report that used invasive arterial pressure measurement to confirm the dramatic decrease in blood pressure in a patient with cough syncope during the Valsalva maneuver and cough induction test without a significant heart rate change. However, further follow‐up on this patient is required.

## AUTHOR CONTRIBUTIONS

**Yoshiro Kubota:** Writing – original draft. **Katsunori Okajima:** Writing – original draft. **Yuichi Nagamatsu:** Writing – original draft. **Tomoyuki Nakanishi:** Writing – original draft. **Takeaki Shirai:** Writing – original draft. **Makoto Kadotani:** Writing – original draft. **Yoshio Ohnishi:** Writing – original draft.

## FUNDING INFORMATION

No financial support was received for this case report.

## CONFLICT OF INTEREST STATEMENT

The authors have no conflicts of interest to declare.

## CONSENT

Written informed consent was obtained from the patient to publish this report in accordance with the journal's patient consent policy.

## Supporting information


Video S1.

## Data Availability

All data generated or analyzed during this study are included in this published article and its supplementary information files.
