# Supplementary material for: A case of cough syncope diagnosed by the Valsalva maneuver and cough induction test under invasive arterial pressure measurement
Source: Clin Case Rep. 2024 Apr 23;12(4):e8798. doi: 10.1002/ccr3.8798 (PMC11039803; doi:10.1002/ccr3.8798)
Supplement: Supplementary file 1 — Video S1. [file CCR3-12-e8798-s001.zip › Caption.docx]

Video S1: A cough induction test using a radial artery line to monitor blood pressure under invasive arterial pressure measurement. After instructing the patient to cough, the systolic blood pressure drastically decreased from 110 mmHg to 50 mmHg with loss of consciousness.
